# Thioredoxin domain-containing protein 9 protects cells against UV-B-provoked apoptosis via NF-κB/p65 activation in cutaneous squamous cell carcinoma

**DOI:** 10.32604/or.2022.028075

**Published:** 2023-03-01

**Authors:** ZHIXUN XIAO, QIUYUN XU, HAIQING WANG, XIAOTONG ZHOU, YANTING ZHU, CHENGBEI BAO, LIHONG CHEN, PENG ZHANG, MIN LIN, CHAO JI, TING GONG

**Affiliations:** 1 Department of Dermatology, The First Affiliated Hospital of Fujian Medical University, Fuzhou, 350000, China; 2 Cancer Center, The First Affiliated Hospital of Fujian Medical University, Fuzhou, 350000, China; 3 Central Laboratory, The First Affiliated Hospital of Fujian Medical University, Fuzhou, 350000, China

**Keywords:** TXNDC9, Ultra Violet-B (UV-B) radiation, Cutaneous squamous cell carcinoma (cSCC), Apoptosis, NF-κB pathway

## Abstract

Cutaneous squamous cell carcinoma (cSCC), a type of non-melanoma skin cancer (NMSC), is the most common malignancy worldwide. Thioredoxin (TXN) domain-containing protein 9 (TXNDC9) is a member of the TXN family that is important in cell differentiation. However, the biological function of this protein in cancer, particularly cSCC, is still unknown. In the present study, our experiments revealed the protective effects of TXNDC9 on UV-B-irritated cSCC cells. The initial findings showed that TXNDC9 is significantly upregulated in cSCC tissue and cells compared to normal skin tissue and keratinocytes. UV-B radiation robustly induces the expression of TXNDC9, and UV-B-induced cSCC cell death is boosted by TXNDC9 deficiency. Moreover, cSCC cells lacking TXNDC9 displayed attenuated activation of the NF-κB pathway. Additional studies by inhibiting TXNDC9 confirmed this finding, as TXNDC9 deficiency attenuated UV-B radiation-induced translocation of NF-κB p65 from the cytoplasm to the nucleus of cSCC. In conclusion, our work demonstrates the biological roles of TXNDC9 in cSCC progression and may provide a novel therapeutic target to treat cSCC in the future.

## Introduction

Cutaneous squamous cell carcinoma (cSCC), a type of non-melanoma skin cancer (NMSC), is one of the most common malignancies worldwide [1]. It causes 20%–30% of keratinocyte carcinomas, affecting more than 3 million people each year. Although mortality is currently declining, cSCC incidence is increasing [2–4]. It has lesions primarily common in the sun-exposure area and would be affected by oxidative stress caused by ultra violet (UV) radiation [5,6]. Over the past 30 years, efforts have been made to understand the numerous interrelated physiological and pathological factors underlying cSCC progression, including genetics, age, oxidative stress, etc. [7–11].

There is a direct relationship between UV radiation from the sun and oxidative damage to skin tissue [12]. Individuals under excessive UV radiation, particularly UV-B (275–320 nm), are at greater risk of skin damage, including solar erythema, photoaging, and even skin cancers [13]. UV-B radiation is known to generate reactive oxygen species (ROS), such as singlet oxygen, superoxide anion, and hydrogen peroxide, leading to a redox imbalance in skin cells [14–16]. However, tumor cells can tolerate stressed environments by upregulating the expression of some antioxidant genes.

The TXN system is a central antioxidant system that protects cells from oxidative stress and helps cells maintain cellular redox homeostasis [17]. Dysfunction of the TXN system can lead to many health problems including metabolic syndrome, cancer, cardiovascular disease, and neurodegeneration [17–19].

Thioredoxin (TXN) domain-containing protein 9 (TXNDC9) is a member of the TXN family that contains the same redox-active site as TXN. Recent studies reported its roles in cell differentiation and prostate cancer progression [20]. However, the biological function of this protein in cancer, particularly cSCC, is still enigmatic.

In the present study, we determined the protective effects of TXNDC9 against UV-B irradiation in cSCC cells. TXNDC9 is significantly upregulated in cSCC tissue and cells compared to normal skin tissue and keratinocytes. UV-B radiation can robustly induce the expression of TXNDC9 in a dose-dependent manner. Moreover, TXNDC9 deficiency was able to augment UV-B-induced cSCC cell death via inhibition of the NF-κB/p65 pathway. In conclusion, our work demonstrates the biological role of TXNDC9 in cSCC progression and may provide a novel therapeutic target to treat cSCC in the future.

## Materials and Methods

### Patients and tissue specimens

Normal human tissue and early cSCC (Stage I and Stage II) sections were obtained from the Department of Dermatology at The First Affiliated Hospital of Fujian Medical University with IRB approval (Suppl. Table 1). This study was approved by the institutional review board of the First Affiliated Hospital of Fujian Medical University (LY2019-013-01). All patients participating in this study received signed written informed consent.

### Cell culture and UV radiation

The HaCaT keratinocyte and cutaneous squamous cell carcinoma cell lines A431 and SCL-1 were purchased from the American Type Culture Collection (ATCC, Manassas, VA) and cultured in Dulbecco’s Modified Eagle’s Medium (DMEM) (Invitrogen Life Technologies, Carlsbad, CA, USA). 10% Fetal Bovine Serum (FBS) (Biomeda, Foster City, CA, USA) and 1% Penicillin/Streptomycin/Glutamine (Gibco/Invitrogen, Carlsbad, CA, USA) in a humidified incubator at 37°C with 5% carbon dioxide. UV-B radiation equipment and procedures were described previously [21–24].

### Antibodies and reagents

The primary antibodies used in this study were rabbit anti-cleaved caspase 3 (1:1000, Cell Signaling Technology, Danvers, MA, USA), mouse anti-β-actin (1:3000, Cell Signaling Technology, Danvers, MA, USA), rabbit anti-NF-κB p65 (1:1000, Cell Signaling Technology, Danvers, MA, USA), rabbit anti-Lamin B1 (1:1000, Cell Signaling Technology, Danvers, MA, USA), and rabbit anti-TXNDC9 (1:200, Abcam, Cambridge, UK). Species-specific secondary antibodies were purchased from Abcam (Cambridge, UK).

### Immunofluorescence

Briefly, xylene dewaxed and alcohol-rehydrated paraffin sections were placed in a bottle filled with a 0.01 M trisodium citrate solution and microwaved. After heating, slides were thoroughly rinsed in cool running water for 5 min. Sections were immersed in 3% H_2_O_2_ at room temperature for 30 min to block any endogenous peroxidase activity. Add rabbit anti-TXNDC9 (Abcam, Cambridge, UK), diluted 1:200 at an appropriate concentration to the tissue, and incubate at room temperature for 2 h, or in a moist chamber overnight. Adding fluorescently conjugated secondary antibodies Goat anti-rabbit IgG H&L (Alexa Fluor 594) (Abcam, Cambridge, UK, ab150080), and incubate for 30 min at room temperature in the dark. Add a small volume of mounting media containing DAPI (Abcam, Cambridge, USA, ab104139) stain and add a coverslip of 15 μl is typically enough to cover a tissue underneath a coverslip fully.

### Immunohistochemistry (IHC)

Briefly, paraffin sections deparaffinized with xylene and rehydrated with alcohol were placed in a bottle containing 0.01 M trisodium citrate solution and heated in a microwave. Then sections were rinsed in cold water for 5 min and soaked in 3% H_2_O_2_ for 30 min at room temperature to block endogenous peroxidase activity. Washed with Tris-buffered saline (TBS) pH 7.4. Then incubated at room temperature for 2 h, or in a moist chamber overnight at 4°C with rabbit anti-TXNDC9 (1:200, Abcam, Cambridge, UK). Sections were then treated with biotin labeled goat anti-rabbit secondary antibodies (Abcam, Cambridge, UK) at 37°C for 30 min, followed by horseradish peroxidase-labeled streptavidin solution. Signals were developed by using diaminobenzidine (DAB) chromogen (Dako, Glostrup, Denmark) as substrate. Meyer's hematoxylin solution (Dako, Glostrup, Denmark) was used as a nuclear neutralizing dye. Incubation without specific antibody was used as a negative control.

### Quantitative PCR

Procedures were described previously [25,26]. Total RNA was isolated using Trizol (Invitrogen Life Technologies, Carlsbad, CA, USA), and cDNA was synthesized using the High-Capacity cDNA Reverse Transcription Kit (Life Technologies/Applied Biosystems, Foster, CA, USA) according to the manufacturer’s instructions.10 ng cDNA was then used in qPCR using SYBR green kits (Thermo Fisher Scientific, Waltham, MA, USA) on a 480 real-time PCR machine (Roche Applied Science, Penzberg, Germany). The specific primers used in qPCR were as follows: primers for TXNDC9 (forward: 5′-CTGCTTCAGACTACCAAACTGG-3′, reverse: 5′-CTCTGTAGAAATGGCAAACCACA-3′) and GAPDH (forward: 5′-ATCAGCAATGCCTCCTGCAC-3′, reverse: 5′-CGTCAAAGGTGGAGGAGTGG-3′). RT-PCR results were calculated using the ∆-Ct method.

### Cell transfection

Methods have been previously described [23,27]. Briefly, short hairpin RNA (shRNA; 5′-TTTGGTAGTCTGAAGCAGC-3′) against TXNDC9 (Sigma–Aldrich, St Louis, MO, USA) and non-target control (NTC; 5′-CACUGAUUUCAAAUGGUGCUAUU-3′) shRNA (Sigma–Aldrich, St Louis, MO, USA) were transfected into cSCC cells using Lipofectamine 2000 Transfection Reagent in accordance to the manufacturer’s instructions. In all studies, cells were transfected 48 h before all treatments.

### Western blot

The methods have been described previously with slight modifications [26,28]. Briefly, whole cell lysates were prepared in cell lysis buffer and clarified by centrifugation. Protein samples of 50 μg were separated on 8%–12% denaturing SDS-PAGE gel (Bio-Rad Laboratories, Hercules, CA, USA) and transferred to polyvinylidene fluoride membranes by electroblotting. Membranes were blocked with blocking buffer (LI-COR, Lincoln, NE, USA) for 30 min at room temperature and then incubated with primary antibodies overnight at 4°C. Blots were incubated with specific secondary antibodies (Abcam, Cambridge, UK) at room temperature for 1 h the next day. The signals were detected by ECL reagents (Meilun Biological Technology, Dalian, China). β-actin (1:3000, Cell Signaling Technology, Danvers, MA, USA) and Lamin B1 (1:1000, Cell Signaling Technology, Danvers, MA, USA) were equivalent loading controls.

### Cell viability assays

Methods have been described previously [25]. Cell viability was assessed by Cell Counting Kit-8 (CCK-8) according to the manufacturer’s instructions (Dojindo Laboratories, Kumamoto, Japan). In brief, When the cells cultured in a 96-well plate grew at a density of 2 × 106/mL, the CCK-8 solution (10 μl) added to each well at 24 h. Then, the absorbance of OD 450 nm was measured by a spectrophotometer (Bio-Rad Laboratories, Hercules, CA, USA).

### Apoptosis assay

The degree of apoptosis in cSCC cells was detected by Western blot analysis of cleaved Caspase-3 and TUNEL assay (Sigma–Aldrich, St Louis, MO, USA). The process has been described [25]. In brief, cells (1 × 106 cells/well) were fixed with 4% paraformaldehyde for 15 min at room temperature and permeabilized with 0.25% Triton X-100 for 20 min at room temperature. Subsequently, after cells were rinsed with PBS, TdT solution and dUTP solution were added and incubated at 37°C for 1 h in the dark. Cells were treated with DAPI (Abcam, Cambridge, USA, ab104139) for nucleus staining for 5 min at 37°C. The cell apoptosis ratio was measured by the TUNEL percentage (TUNEL/Hoechst 33342 × 100%). The results shown were representative of at least 200 cells in 5 random scope fields per treatment.

### NF-kB DNA binding assay

The activation of NF-κB used NF-κB (p65) Transcription Factor Assay Kit (Cayman Chemical, Ann Arbor, Michigan, USA) that detects specific NF-κB (p65) DNA binding activity in nuclear extracts. In this study, 10 μg of nuclear fractions extracted from cSCC cells were run in duplicate along with positive and negative controls. After incubation with the primary and secondary antibody, add developing solution and measure sample at an absorbance at 450 nm using a microplate reader (Bio-Rad Laboratories, Hercules, CA, USA).

### Statistical analysis

All data shown in this study were expressed as the mean values ± SEM of at least three independent experiments. Appropriate statistical tests were performed using GraphPad Prism 7.0 software to calculate *p*-values (GraphPad Software Inc., San Diego, CA). A significant difference considered to be present at *p* < 0.05.

## Results

### TXNDC9 expression is upregulated in cSCC tissue and cell lines

**Figure 1:**
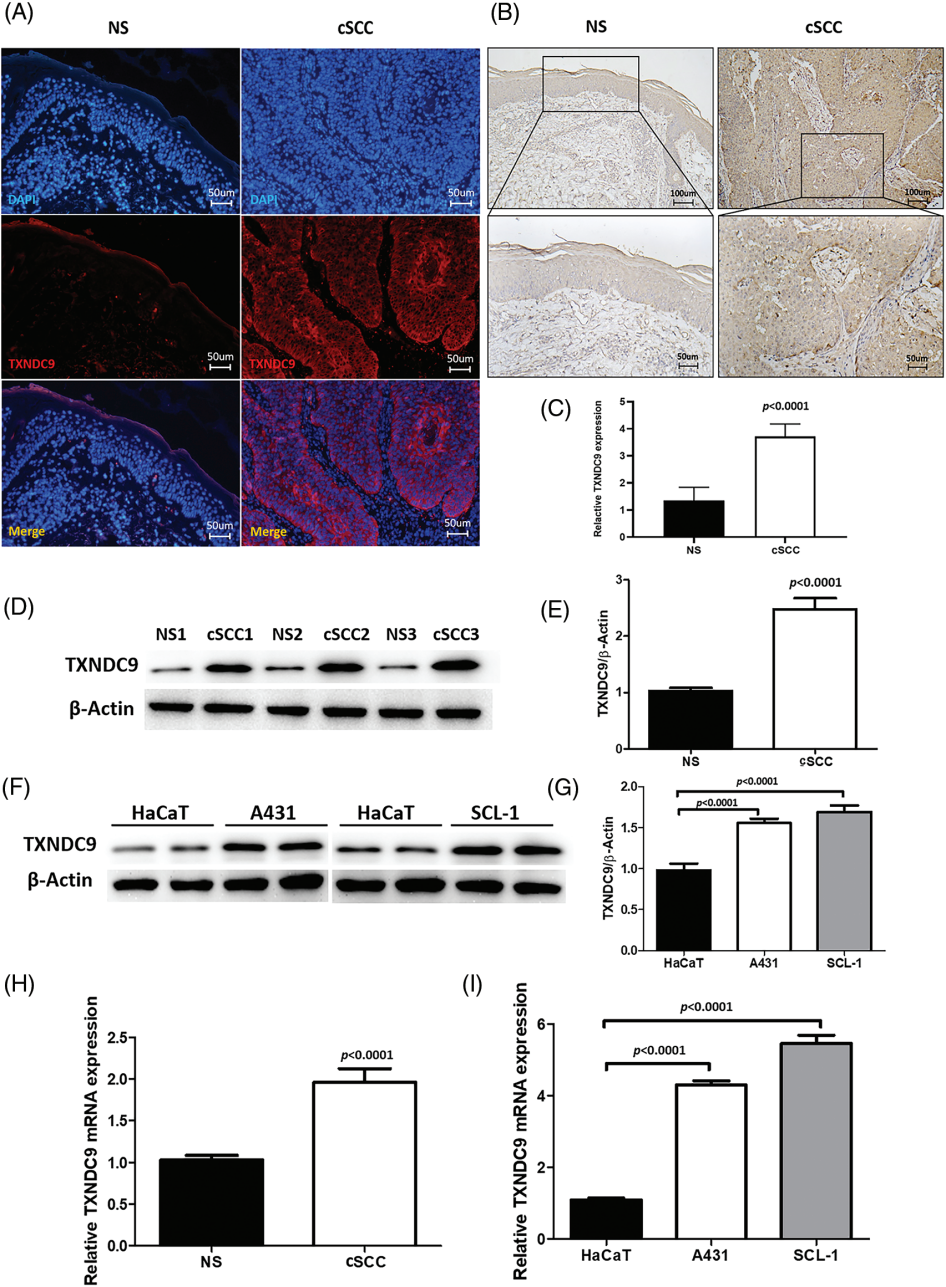
Upregulation of TXNDC9 levels is detected in cSCC tissues and cell lines. (A) Immunofluorescence staining of human normal skin (NS) and cSCC samples (original magnification X200). Tissues were stained for TXNDC9 (Red) and DAPI (Blue). (B) Immunohistochemistry staining of TXNDC9 in NS and cSCC samples (original magnification X100, X200). (C) Quantification of TXNDC9 levels in NS and cSCC samples. (D) Western blot analysis of TXNDC9 in NS and cSCC samples from three patients. (E) TXNDC9 levels were quantified. (F) TXNDC9 protein levels of HaCaT cells and cSCC cells (A431 and SCL-1) were detected by western blot. (G) TXNDC9 levels of cSCC cells were quantified. mRNA levels of TXNDC9 were assessed by RT-PCR in (H) tissues and (I) cSCC cell lines.

**Supplementary Figure 1:**
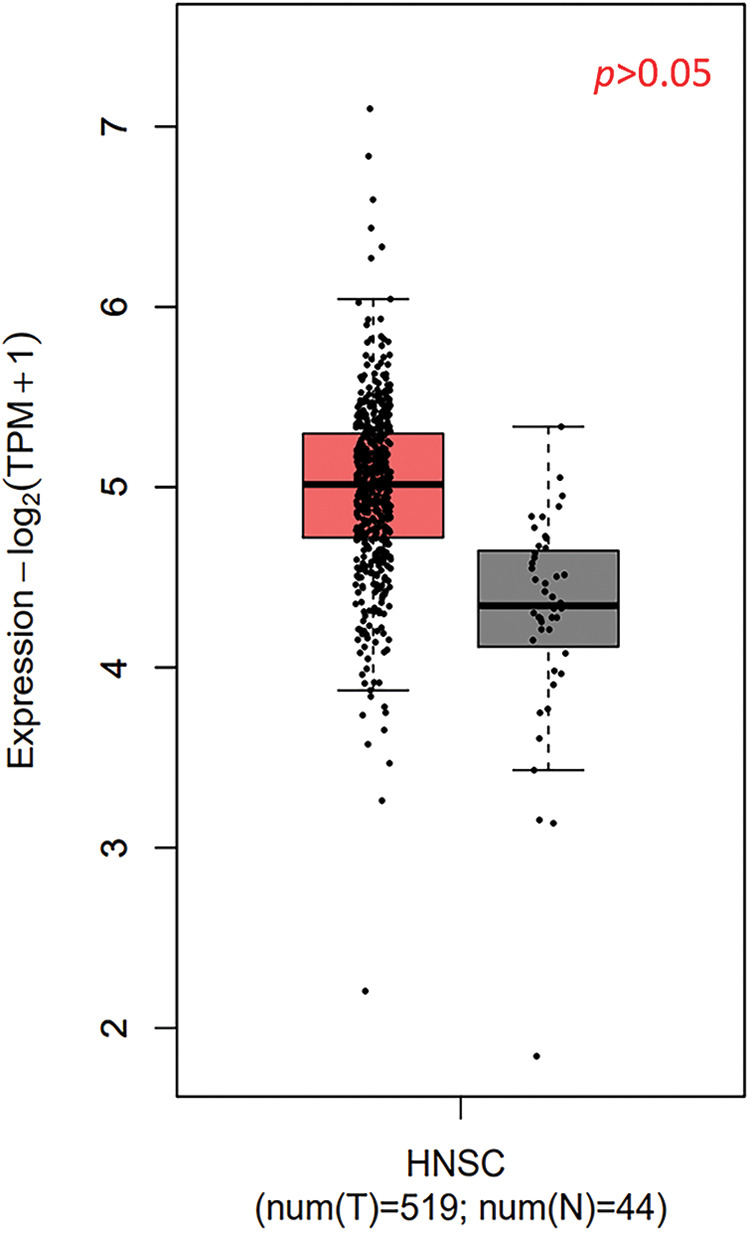
The expression of TXNDC9 in head and neck squamous cell carcinoma (HNSC) and normal tissues using datasets from The Cancer Genome Atlas (TCGA) database.

TXNDC9 is a critical regulator in cell proliferation in some types of cancers [20,29]. To identify the role of TXNDC9 in cSCC, we first conducted a series of experiments to examine the expression of TXNDC 9 in cSCC tissues and cell lines. Our results demonstrated that TXNDC9 protein levels were significantly higher in cSCC tissue samples than in standard skin samples (Figs. 1A–1E). Moreover, TXNDC9 mRNA levels were then assessed by RT-PCR and observed to be upregulated (Fig. 1H). Consistent with the results of tissue samples, TXNDC9 expression was robustly elevated in two cSCC cell lines, A431 and SCL-1, as well (Figs. 1F, 1G and 1I). These results revealed that TXNDC9 expression is significantly higher in cSCC than in normal keratinocytes and skin tissues, suggesting a positive correlation between TXNDC9 and cSCC progression. In addition, we analyzed the expression of TXNDC9 in head and neck squamous cell carcinoma (HNSC) and normal tissues using datasets from The Cancer Genome Atlas (TCGA) database (Suppl. Fig. 1).

### UV-B radiation stimulates the induction of TXNDC9 expression

**Figure 2:**
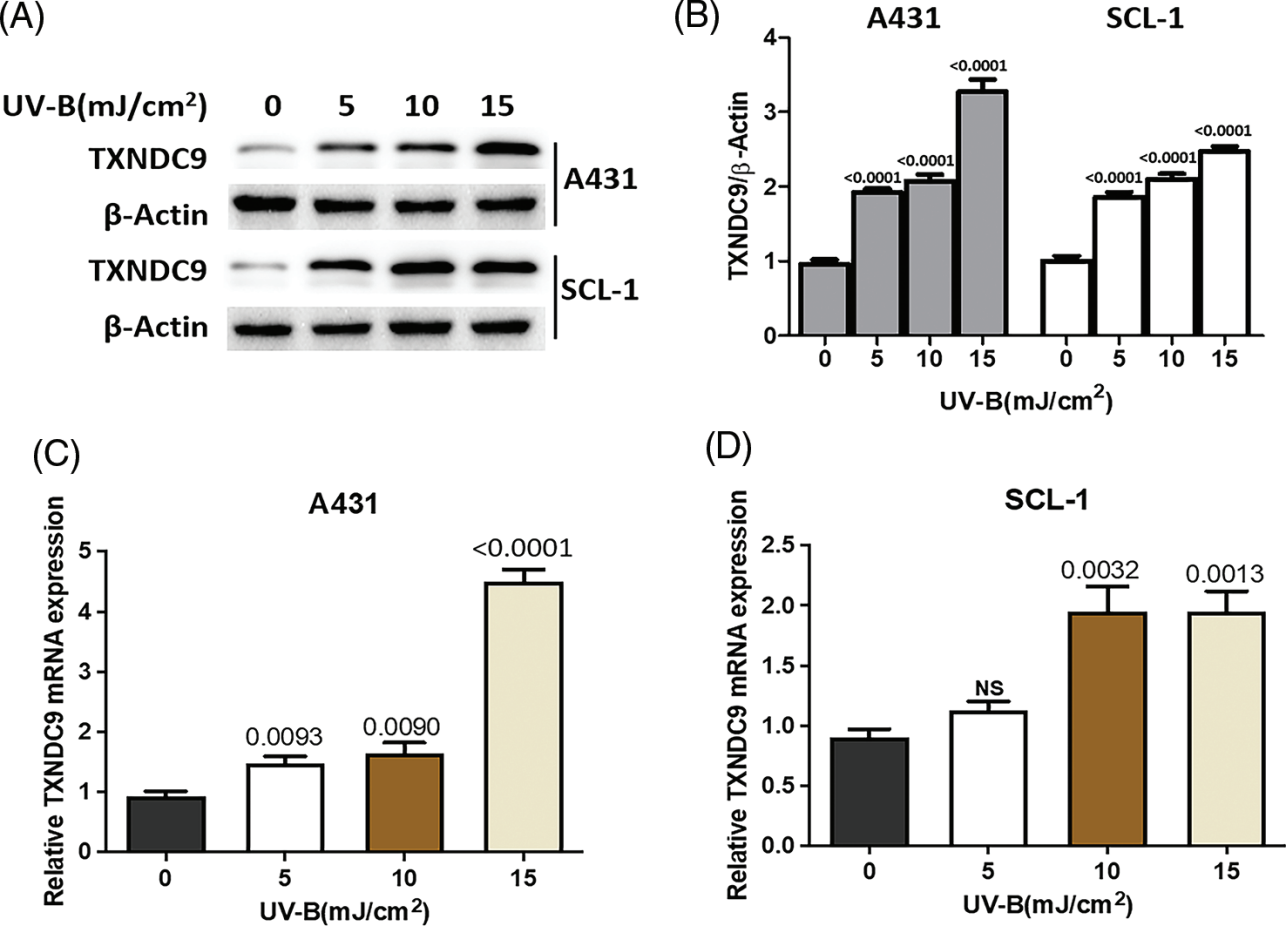
UV-B radiation induces TXNDC9 levels in cSCC cell lines. A431 and SCL-1 cells were subjected to UV-B radiation at indicated dose. (A) After 24 h, TXNDC9 levels were examined by western blots. (B) Protein expressions were quantified by Image J software. mRNA levels of TXNDC9 were also assessed by RT-PCR in (C) A431 and (D) SCL-1 cells. “NS” stands for “non-significant”.

Next, we tested the potential effect of UV-B in TXNDC9 expression. A431 and SCL-1 cells were subjected to different UV-B radiation intensities (0, 5, 10, 15 mJ/cm^2^). After 24 h, TXNDC9 protein levels were examined by Western blots analysis. Results revealed that protein levels of TXNDC9 were robustly induced by UV-B radiation in cSCC cells in a dose-dependent manner (Figs. 2A and 2B). Subsequent analysis by RT-PCR in both cell lines showed that TXNDC9 expression was also induced mainly in response to UV-B radiation (Figs. 2C and 2D). These results suggest that TXNDC9 expression is positively correlated with UV-B radiation.

### TXNDC9 deficiency augments UV-B-provoked cell death in cSCC cells

**Figure 3:**
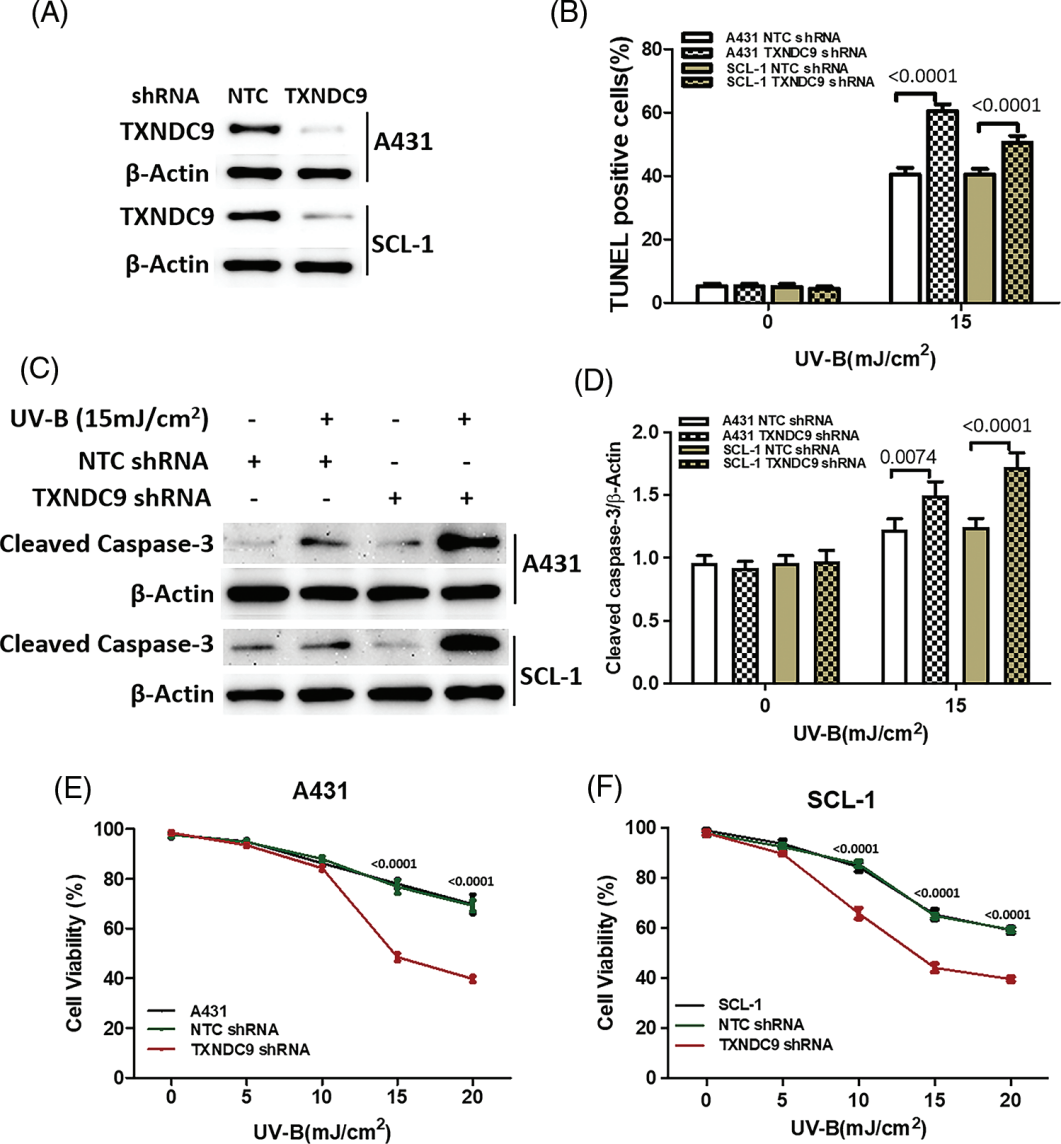
TXNDC9 protects cSCC cells against UV-B radiation induced cell death in a dose dependent manner. cSCC cells were first transfected with NTC or TXNDC9 shRNA prior to the UV-B radiation. These cells were then cultured in complete medium for additional 24 h. (A) Expression of TXNDC9 after transfection was assessed by western blot. (B) Apoptotic cells were measured by fluorescent TUNEL assay. (C) Apoptosis levels in cSCC cells were analyzed by measuring cleaved caspase-3 levels using western blot. (D) Cleaved caspase-3 levels were elevated when knocking down TXNDC9. Cell viability of (E) A431 and (F) SCL-1 cells were tested by MTT assay.

**Supplementary Figure 2:**
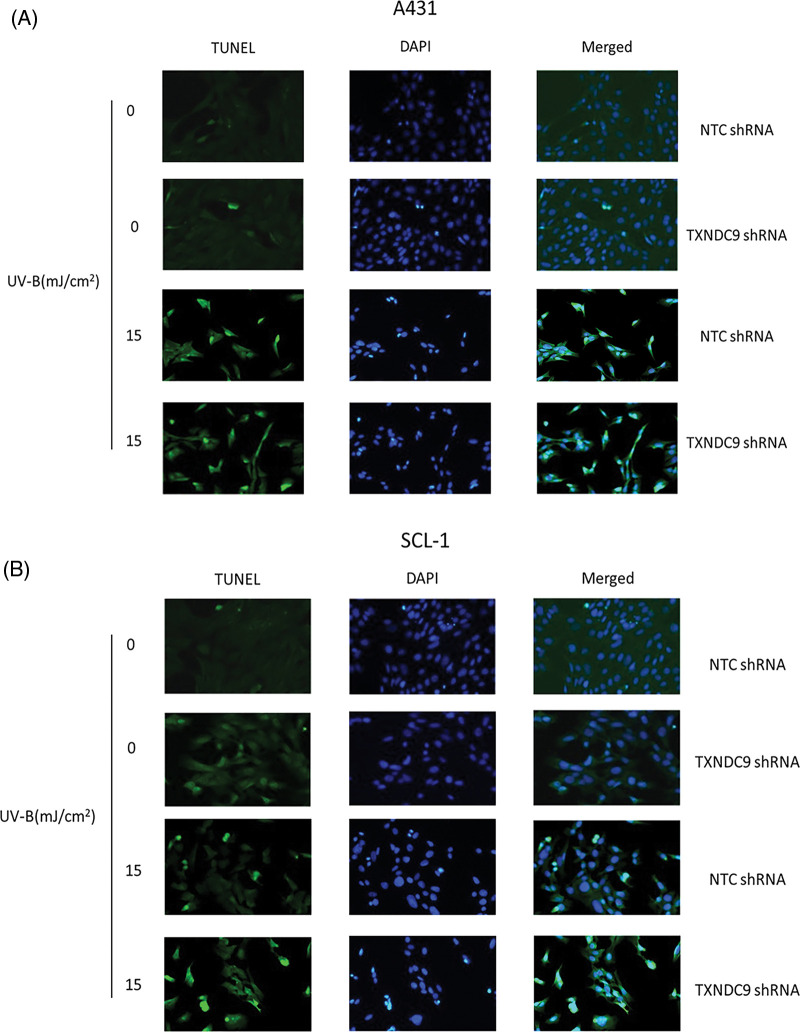
Apoptotic cells of (A) A431 and (B) SCL-1 cells were measured by fluorescent TUNEL assay.

To further define the biological effect of TXNDC9 in cSCC cell progression and its underlying mechanism, the shRNA method was applied to knock down TXNRD9 in cSCC cells. Western blot analysis showed that TXNDC9 shRNA effectively downregulated TXNDC9 in both cSCC cell lines compared to NTC shRNA (Fig. 3A). Next, we examined the degree of UV-B-induced cell apoptosis using TXNDC9-knockdown cSCC cells. UV-B-provoked cell apoptosis was tested by TUNEL staining assay (Figs. 3B, Suppl. Figs. 2A and 2B) and Western blot analysis of cleaved caspase-3 levels (Figs. 3C and 3D). Results from these assays supported that UV-B radiation-induced apoptosis in cSCC cells and that this effect was significantly provoked by TXNDC9 deficiency. By these results, MTT cell survival assay revealed that UV-B radiation decreased cSCC cell survival, which was promoted mainly when TXNDC9 was silenced (Figs. 3E and 3F). The evidence suggests that TXNDC9 mediates a cytoprotective effect in cSCC cells against UV-B.

### NF-κB/p65 activation mediates TXNDC9-induced cytoprotection against UV-B radiation

**Figure 4:**
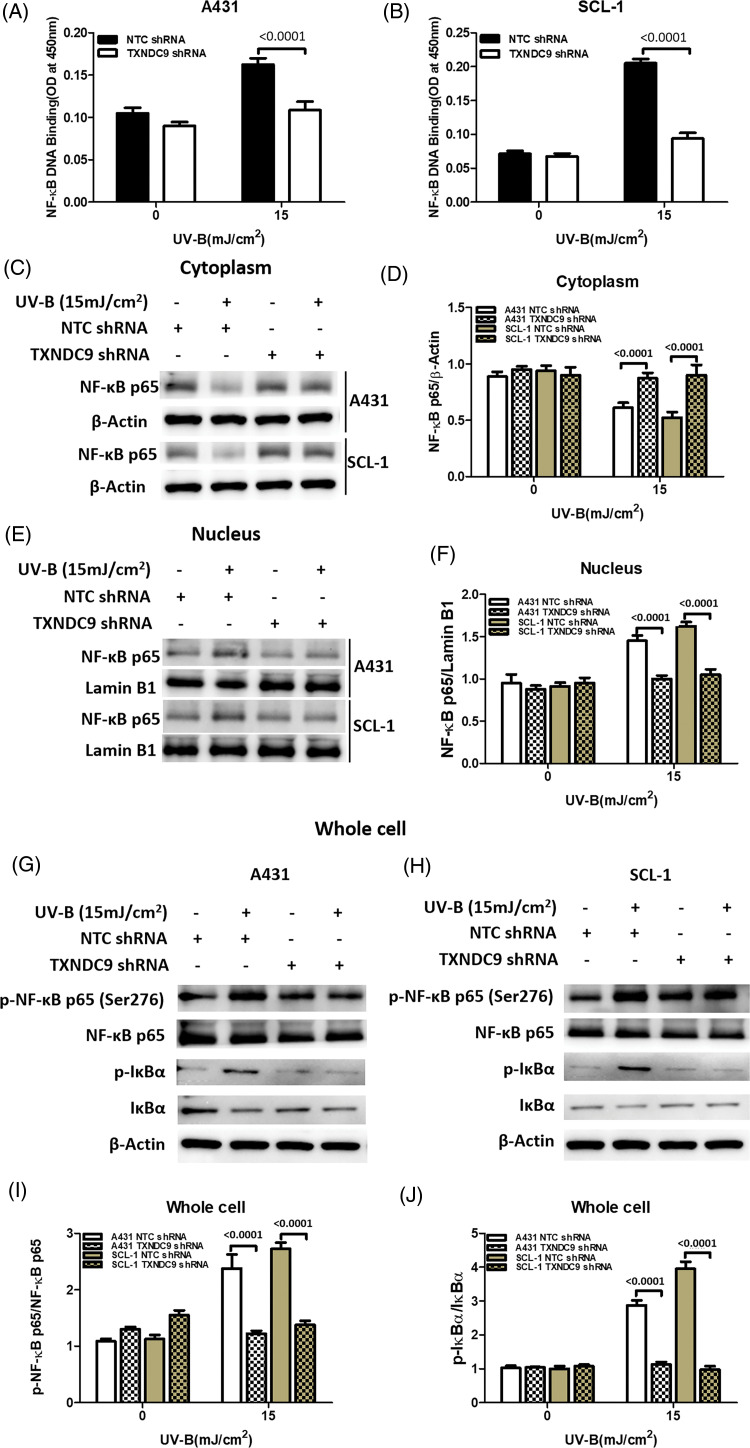
TXNDC9 deficiency impairs UV-B-induced NF-κB/p65 activation. cSCC cells were first transfected with NTC or TXNDC9 shRNA prior to UV-B irradiation. Cells were cultured for an additional 24 hours in incomplete medium. (A) & (B) UV-B radiation induced NF-κB binding activity in cSCC cells, and TXNDC9 deletion significantly reduced NF-κB binding activity. NF-κB p65 immunoblot analysis in (C) the cytoplasm, (E) the nuclear, and (G) & (H) whole cell lysates. (D) Quantitative western blot of cytoplasm results showed that UV-B radiation inhibited NF-κB p65, but it was significantly increased in TXNDC9-deficient cells. (F) Quantitative western blot of nuclear results showed that UV-B radiation elevated NF-κB p65, but it was significantly repressed in TXNDC9-deficient cells. (I) & (J) Phosphorylation of NF-κB p65 and IκBα, total NF-κB p65, and IκBα in the whole cell lysates were quantified by densitometry.

**Supplementary Figure 3:**
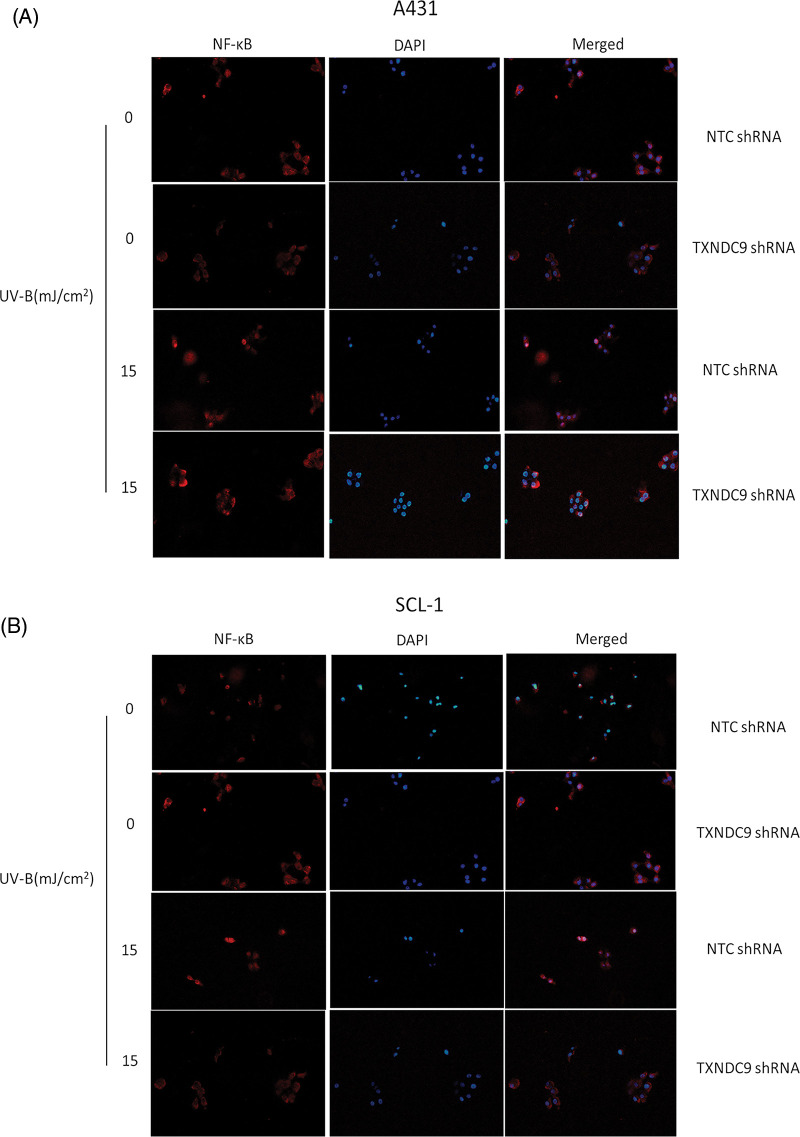
Immunofluorescence of NF-κB p65 and DAPI in (A) A431 and (B) SCL-1 cells’ nucleus after UV-B.

Our previous results above showed that TXNDC9 promotes cell progression in cSCC cells. To address the underlying mechanism of TXNDC9-mediated cSCC cell survival, we performed a set of studies to verify whether this biological function is related to NF-κB activation since the NF-κB pathway plays a critical role in many biological signs of progress. TXNDC9-deficient A431 and SCL-1 cells were challenged with UV-B radiation, and examined NF-κB DNA binding activity. TXNDC9-deficient cSCC cells showed significantly lower NF-κB activation (Figs. 4A and 4B). In addition, proteins extracted from the cytoplasm (Figs. 4C and 4D), nucleus (Figs. 4E and 4F), and whole cells were also subjected to western blotting to detect proteins of the NF-κB-p65 signaling pathway. Loss of TXNDC9 significantly attenuates UV-B radiation-induced translocation of NF-κB p65 from the cytoplasm to the nucleus of cSCC (Figs. 4G–4I). Moreover, phosphorylation of NF-κB p65 and IκBα induced by UV-B radiation were also attenuated by TXNDC9 deficiency (Figs. 4G, 4H and 4I). In addition, Immunofluorescence of NF-κB p65 and DAPI in the nucleus after UV-B has evidenced the relationship between TXNDC9 and NF-κB signaling (Suppl. Fig. 3). These results show that NF-κB/p65 activation is critical for TXNDC9-induced cytoprotection against UV-B radiation in cSCC cells.

## Discussion

The prevalence of cSCC is rising rapidly and broadly in correlation to excessive UV radiation and photooxidative stress [12–14]. A growing interest exists in novel strategies for the diagnosis, treatment, and prevention of cSCC. We demonstrate TXNDC9 as a potent antioxidant protein that protects cSCC cells from UV-B-induced oxidative damage.

Excessive UV-B radiation is the fundamental environmental risk factor that underlies skin damage [30–32]. However, in tumorous microenvironments, cancer cells will develop their counterplan to confront different stress and survive. Particularly under chronic oxidative stress, increased levels of antioxidant proteins are detected and are observed to promote cell growth and tumor progression [33]. TXNs contains a redox-active site (CXXC) and are kept in reduced form to regulate redox homeostasis [34]. TXN system is implicated in many biological processes, including cell proliferation and apoptosis [35]. Therefore, this redox system has become a focus of interest in various cancer types [36]. TXN1 has been found to be upregulated in several cancers, including prostate, colon and gastric cancer, to promote cancer progression and predict poor prognosis [37–39]. Thioredoxin domain containing 5 (TXNDC5) is also implicated in the regulation of cancer progression of hepatocellular carcinoma, prostate cancer, and colorectal cancer [40–42]. TXNDC9 is involved in cancer progression as well. Feng et al. reported that TXNDC9 is a crucial regulator of cell survival and proliferation in prostate cancer and is associated with advanced clinical stages [43]. In hepatocellular carcinoma, recent studies have demonstrated that TXNDC9 promotes cancer progression by mediating the gene regulation networks [29]. However, the role of TXNDC9 in cSCC remains unknown. In our study, we first found that TXNDC9 is significantly upregulated in cSCC tissue and cells. Moreover, UV-B radiation can robustly induce the expression of TXNDC9. The UV-B-induced photooxidative damage is significantly augmented by the inhibition of TXNDC9. Our findings establish TXNDC9 as a positive regulator in cSCC development and demonstrate the potentially use of TXNDC9 as a therapeutic target or biomarker in cSCC patients.

To further investigate the association between TXNDC9 and cSCC, we performed a series of studies using TXNDC9 deficient cSCC cells to assess potential affected cellular signaling pathways under UV-B radiation. An enormous amount of literature has recently accumulated indicating that many thioredoxin family members are critical to regulating the DNA-binding activity of transcription factors, including NF-κB [44,45]. TXNs activate NF-κB signaling by reducing cysteine residues in p50/p65 through its active redox site [44,45]. Our present studies add more evidence in support of this notion. Herein, we demonstrated TXNDC9 regulation of NF-κB DNA binding activity induced by UV-B radiation. The NF-κB/p65 activation is documented to be critical for many biological processes, such as inflammation, cell survival and proliferation, neuronal survival, metastasis, and so on [46,47]. Regarding its specific role in cell cycle regulation, NF-κB activation is observed in many cancers and is potent in promoting cell survival and proliferation [46–48]. In the present study, we verified that TXNDC9 deficiency inhibits NF-κB/p65 activation and translocation from cytoplasm to nucleus induced by UV-B-radiation. The data presented herein add to an accumulating body of evidence demonstrating that NF-κB activation is a critical regulatory pathway in cancer development. More importantly, our study has linked TXNDC9 with the NF-κB signaling pathway for the first time and determined the underlying mechanism of TXNDC9-mediated cSCC progression. Therefore, TXNDC9-regulated NF-κB/p65 activation could be a prospective target in the treatment of cSCC.

Our results show that TXNDC9 is upregulated in cSCC tissues and protects cSCC cells from UV-B radiation-mediated apoptosis via activating NF-κB signaling. TXNDC9 could serve as a potential and promising therapeutic target in cSCC treatment.

## Data Availability

The data and materials used in the current study are available from the corresponding author on reasonable request.

## Appendix

**Supplmentary Table 1:** Demographic characteristics of 32 CSCC patients and 34 normal controls

	No. of cases	No. of controls	χ^2^ *p* value*
Total	32	34	
Age, y			
30–60	14	19	0.32
60–90	18	15	
Sex			
Male	20	20	0.76
Female	12	14	
TMN stage			
I/II	32	Not applicable	
III/IV	0	Not applicable	

Note: *Comparision between each variable was calculated with Chi-square test.
